# Metaproteomics Reveals Alteration of the Gut Microbiome in Weaned Piglets Due to the Ingestion of the Mycotoxins Deoxynivalenol and Zearalenone

**DOI:** 10.3390/toxins13080583

**Published:** 2021-08-21

**Authors:** Johan S. Saenz, Alina Kurz, Ursula Ruczizka, Moritz Bünger, Maximiliane Dippel, Veronika Nagl, Bertrand Grenier, Andrea Ladinig, Jana Seifert, Evelyne Selberherr

**Affiliations:** 1 Institute of Animal Science, University of Hohenheim, Emil-Wolff-Str. 6-10, 70593 Stuttgart, Germany; alina.renz@uni-hohenheim.de; 2 HoLMiR—Hohenheim Center for Livestock Microbiome Research, University of Hohenheim, Leonore-Blosser-Reisen Weg 3, 70593 Stuttgart, Germany; 3 University Clinic for Swine, University of Veterinary Medicine Vienna, Veterinaerplatz 1, 1210 Vienna, Austria; ursula.ruczizka@vetmeduni.ac.at (U.R.); moritz.buenger@vetmeduni.ac.at (M.B.); maximiliane.dippel@vetmeduni.ac.at (M.D.); andrea.ladinig@vetmeduni.ac.at (A.L.); 4 BIOMIN Research Center, Technopark 1, 3430 Tulln, Austria; veronika.nagl@dsm.com (V.N.); bertrand.grenier@dsm.com (B.G.); 5 Institute of Food Safety, Food Technology and Veterinary Public Health, Unit of Food Microbiology, University of Veterinary Medicine Vienna, Veterinaerplatz 1, 1210 Vienna, Austria; evelyne.selberherr@vetmeduni.ac.at

**Keywords:** mycotoxins, metaproteomics, deoxynivalenol, zearalenone, gut microbiome

## Abstract

The ingestion of mycotoxins can cause adverse health effects and represents a severe health risk to humans and livestock. Even though several acute and chronic effects have been described, the effect on the gut metaproteome is scarcely known. For that reason, we used metaproteomics to evaluate the effect of the mycotoxins deoxynivalenol (DON) and zearalenone (ZEN) on the gut microbiome of 15 weaned piglets. Animals were fed for 28 days with feed contaminated with different concentrations of DON (DONlow: 870 μg DON/kg feed, DONhigh: 2493 μg DON/kg feed) or ZEN (ZENlow: 679 μg ZEN/kg feed, ZENhigh: 1623 μg ZEN/kg feed). Animals in the control group received uncontaminated feed. The gut metaproteome composition in the high toxin groups shifted compared to the control and low mycotoxin groups, and it was also more similar among high toxin groups. These changes were accompanied by the increase in peptides belonging to Actinobacteria and a decrease in peptides belonging to Firmicutes. Additionally, DONhigh and ZENhigh increased the abundance of proteins associated with the ribosomes and pentose-phosphate pathways, while decreasing glycolysis and other carbohydrate metabolism pathways. Moreover, DONhigh and ZENhigh increased the abundance of the antioxidant enzyme thioredoxin-dependent peroxiredoxin. In summary, the ingestion of DON and ZEN altered the abundance of different proteins associated with microbial metabolism, genetic processing, and oxidative stress response, triggering a disruption in the gut microbiome structure.

## 1. Introduction

Food safety issues are rising due to the increase in the world population and the higher demand for food production. Among those issues, food crop contamination with mycotoxins has gained attention in the last few years. It was estimated that approximately 20% of cereals and nuts are contaminated with mycotoxin levels exceeding the regulatory limits of the EU and Codex Alimentarius standards. However, detectable concentrations of mycotoxins can be found in 60%–80% of samples [1]. Additionally, a long-term study across 100 countries found that 88% of the feed is contaminated with at least one mycotoxin [2].

Mycotoxins are secondary metabolites of low molecular weight typically produced by filamentous fungi [3] such as *Aspergillus*, *Penicillium*, *Alternaria*, and *Fusarium* [4]. These secondary metabolites are difficult to classify and define due to their diverse chemical structure, origin, and effect. Despite the diversity and prevalence of mycotoxins, only a small group of them are recognized as a food, health and economic hazard [5], among which are aflatoxins, ochratoxins, fumonisins, zearalenone and trichothecenes [5,6,7]. Mycotoxins accumulate in both pre- and post-harvest processes and resist different food processing methods, which limits their removal from the food chain [8,9,10,11]. Humans are exposed to mycotoxins by direct plant consumption; however, products of animal origin, such as milk, also represent a risk [12]. Deoxynivalenol (DON), fumonisin and zearalenone (ZEN) are the most prevalent mycotoxins found in maize and wheat, which are commonly used for livestock feeding [2,6]. The high co-occurrence of these mycotoxins could indicate their involvement in synergistic and additive interactions [5,13].

Mycotoxins can have different carcinogenic, hepatotoxic, teratogenic, and mutagenic effects on humans and animals [10,14]. Moreover, some mycotoxins exert a negative effect on nutrient absorption and the barrier function of the gastrointestinal tract [13]. These changes seem to alter the gut microbiome and digesta composition, biotransformation of xenobiotics as well as susceptibility to infections [15]. ZEN and DON alter the gut microbial diversity, more specifically phyla such as Firmicutes, Bacteroidetes, Proteobacteria, and Actinobacteria [13,15]. Although the effect of the toxins varies depending on the age of the animal, diet, time, dosage, and gut section [13,15], most of the available studies describe the effect of the toxins on the microbial diversity and not their functional protein profile.

In this study, we evaluated the effect of two mycotoxins (DON and ZEN) commonly found in livestock feed on the gut metaproteome of 15 weaned piglets. The objective of the study was to evaluate the alteration of the microbial composition and function in the small intestine of weaned piglets after the ingestion of different dosages of the mycotoxins.

## 2. Results

### 2.1. Effect of the Mycotoxins on the Animal Health

**Figure 1:**
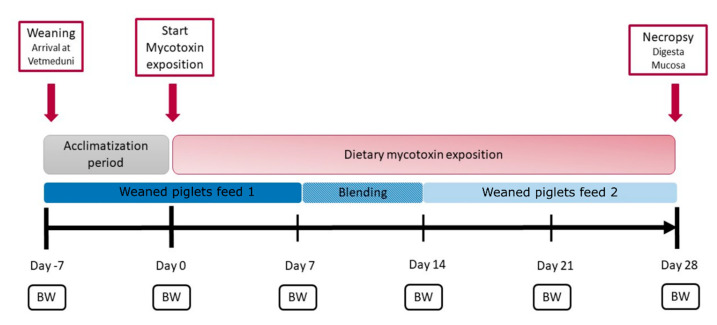
Timeline of the animal experiment. After arrival at the Vetmeduni Vienna on day 7, all weaned piglets started an 8-day acclimation phase, in which the feed administered to the control group was fed to all piglets. Mycotoxin exposition started on day 1, and weaned piglets were fed with mycotoxin-contaminated feed according to their groups. Control weaned piglets received control feed without mycotoxins during this period. Weaned piglets feed 1 and weaned piglets feed 2 were used as base diets for all groups. Body weight (BW) was measured weekly.

The effects of mycotoxin-contaminated diets on performance parameters of piglets are reported in detail by Bünger et al. (2020) [16]. Briefly, independent of the used contamination level, neither DON nor ZEN had an influence on the body weight of weaned piglets. Clinical signs of DON-mycotoxicosis, such as feed refusal or vomiting, were absent. Additionally, ZEN exposure caused signs of hypoestrogenism in the weaned piglets, such as enlargement of the vulva (Figure 1).

### 2.2. Effect of the Mycotoxins on the Global Microbial Protein Composition

**Figure 2:**
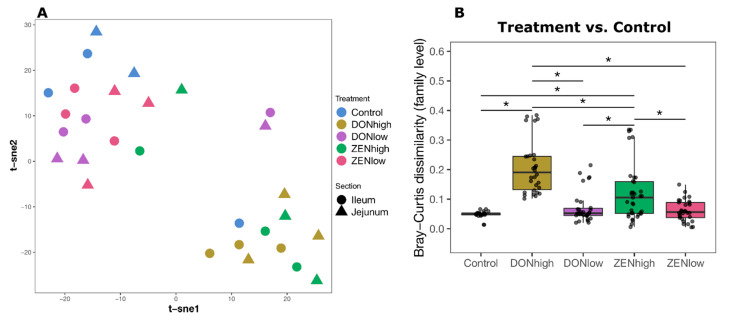
The gut metaproteomic composition is altered by DONhigh and ZENhigh. (**A**) Unsupervised dimensionality reduction analysis for the digesta samples (Perplexity = 10, maximum iteration = 1200). Clustering by treatment (*p* = 0.001, PERMANOVA permutations = 999) and intestinal section (*p* = 0.076, permutations = 999). (**B**) Bray–Curtis dissimilarity (Control vs. Treatment) of peptides identified at the family level. * *p* < 0.05 Kruskal–Wallis and Wilcoxon rank sum test, *p* values were corrected by Benjamini–Hochberg for multiple comparisons.

In total, 17,202 peptide sequences corresponding to 2094 protein groups were identified across all samples. Out of the total identified protein groups, 76% corresponded to the gut microbiome and 24% to the host. A larger number of those protein groups were identified in the digesta samples compared to the mucosa. Additionally, 88% and 34% of the identified protein groups in digesta and mucosa, respectively, belonged to the gut microbiome (Table S1). Only 165 protein groups were identified in the microbiome–mucosa fraction, which was not enough to perform subsequent analysis. Identified host proteins did not show differences between the treatments. The variance associated with the treatments and the gut section was evaluated using a 2-way PERMANOVA test based on Bray–Curtis dissimilarities. Digesta samples were significantly clustered according to the treatment (*p* = 0.001, permutations = 999) and not according to the intestinal section (*p* = 0.076, permutations = 999). Additionally, a different effect of the treatment on the intestinal section was not found (interaction *p* = 0.954, permutations = 999). The treatment and intestinal section categories explain 28.7% and 13.7% of the variability between the samples, respectively. An unsupervised non-linear dimensionality reduction algorithm, t-distributed stochastic neighbor embedding (tSNE), was used to visualize the clustering of the samples (Figure 2A). Global microbial protein composition was affected by DONhigh and ZENhigh in the digesta samples. Samples from DONhigh and ZENhigh clustered together, while DONlow and ZENlow clustered with the control group. As suggested by the previous PERMANOVA analysis, a clustering due to the gut sections was not evident. Subsequently, samples from both the jejunum and ileum were merged and used in the downstream analysis. Next, the Bray–Curtis dissimilarity of the peptides identified at the family level of each treatment compared to control was calculated (Figure 2B). The Bray–Curtis dissimilarity between the treatments and control indicated that DONhigh and ZENhigh significantly shifted the microbiome at the family level (Pair Wilcox test, BH adjusted, *p* < 0.05). No such effects were found for the DONlow and ZENlow groups.

### 2.3. Effect of the Mycotoxins on Microbial Composition

**Figure 3:**
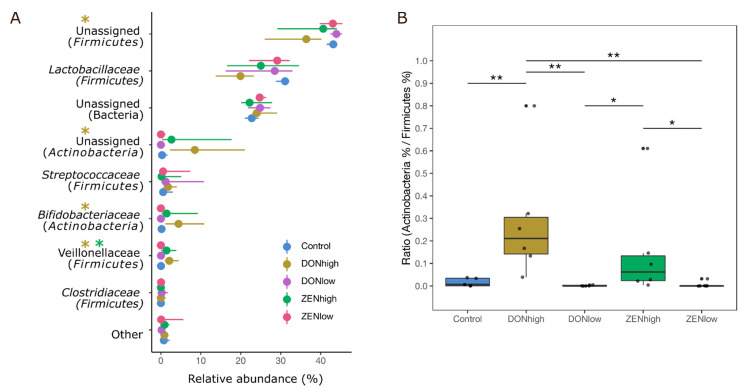
DONhigh and ZENhigh increased the relative abundance of Actinobacteria and decreased the abundance of Firmicutes. (**A**) Relative abundances of the most abundant bacterial families across all treatments. * Significant difference between the treatment and control group. 95% confidence interval. (**B**) Ratio between the relative abundance of the phyla Actinobacteria and Firmicutes. *p* < * 0.05, ** 0.005, Kruskall-Wallis and Wilcoxon rank sum test, *p* values were corrected by Benjamini-Hochberg for multiple comparisons.

The effect of the mycotoxins on the microbiome composition based on the calculated relative abundance of peptide intensity associated with different taxa was examined. Between 22.8% and 24.8% of the peptides were classified only as Bacteria or Archaea. In general, Firmicutes (59.8%–75.1%) was the most abundant phylum across the samples, followed by Actinobacteria (0.1%–14.9%) and Proteobacteria (0.05%-0.4%) (Figure S1, Table S2). DONhigh and ZENhigh changed the relative microbiome composition. DONhigh decreased the relative abundance of Firmicutes and Planctomycetes compared to the control group, while Actinobacteria increased (*p* < 0.005). ZENhigh showed a similar trend, but it was not significant compared to the control group (*p* > 0.05, Table S3). Relative abundance of Firmicutes decreased 0.2- and 0.1-fold in DONhigh and ZENhigh, respectively, compared to the control group, while Actinobacteria increased 12.5- and 6.9-times, respectively (Figure S1). Plantomycetes and Cyanobacteria were found in minor abundance only in the control group. Additionally, Euryarchaeota and unclassified Archaea were not detected in ZENhigh samples, but the abundance of these groups was highly variable across the metaproteomes (Table S2). At the family level, eight families out of the total of 36 bacterial and archeal families found were significantly different between the treatments (Figure 3A). *Bifidobacteriaceae* were less abundant in the control group (0.43%) and increased in DONhigh and ZENhigh (2.8%–5.1%). In contrast, *Lactobacillaceae,* a highly abundant family in the control group (30.7%), decreased in DONhigh and ZENhigh samples (19.1–25.1%). Lowly abundant Firmicutes families found in the control animals such as *Acidaminococcaceae*, *Veillonellaceae* and *Selenomonadaceae* significantly increased between 3- and 60-fold in DONhigh and ZENhigh (Figure 3A). In general, no or only a low effect was observed in the microbial composition of DONlow and ZENlow. The calculated ratio of Actinobacteria/Firmicutes across the samples showed that DONhigh and ZENhigh had a significant effect on the dominance of Firmicutes compared to Actinobacteria (Figure 3B). A decrease in the abundance of peptides assigned to Firmicutes was always accompanied by an increase in Actinobacteria-related peptides. The relative abundance of Actinobacteria increased by between 8.2% and 15.1% in the animals of the DONhigh and ZENhigh groups.

### 2.4. Effect of the Mycotoxins on Gut Microbiome Function

**Figure 4:**
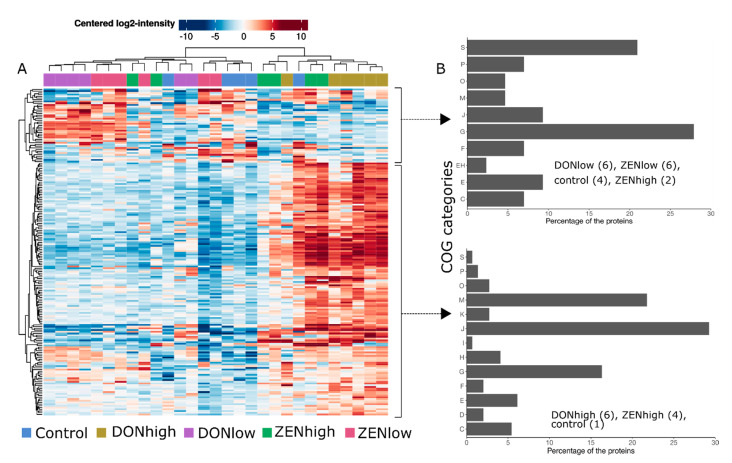
Bacterial protein profiles were more similar between DONhigh and ZENhigh. (**A**) Clustering of the 190 significantly different proteins group across the treatments. *p* < 0.05, Log2 fold change > 2, and >1 peptides identified. (**B**) The number of proteins identified by COG categories for each cluster of protein groups. (J) Translation, ribosomal structure and biogenesis, (G) carbohydrate transport and metabolism, (M) cell wall/membrane/envelope biogenesis, (S) function unknown, (E) amino acid transport and metabolism, (C) energy production and conversion, (F) nucleotide transport and metabolism, (H) coenzyme transport and metabolism, (O) post-translational modification, protein turnover, and chaperones, (K) transcription, (D) cell cycle control, cell division, chromosome partitioning, (P) inorganic ion transport and metabolism, (L) replication, recombination and repair, (I) lipid transport and metabolism.

Differentially abundant protein groups and samples were compared and clustered using LFQ-Analyst. Transformed LFQ intensities of the retained proteins were compared between the treatments. One hundred and ninety protein groups, 28.1% of the total evaluated, were significantly different across treatments (Figure 4A). The protein groups were classified in COG (Clusters of Orthologous Groups of proteins) categories. Most of the proteins were associated with translation, ribosomal structure, and biogenesis (25.4%), followed by carbohydrate transport and metabolism (19.4%), and cell wall/membrane/envelope biogenesis (18.3%) (Figure S2). Based on the differentially abundant protein profiles, all samples from DONhigh clustered together, as well as some samples from ZENhigh (Figure 4A). This indicates that the gut microbiome protein profile is more similar between the animals that received DONhigh and ZENhigh (Figure S3). Differentially abundant proteins clustered in two groups based on their intensity (Figure 4A). The first cluster, composed of 43 proteins, was enriched in DONlow, ZENlow and control samples, while the second cluster, composed of 147 proteins, was enriched in DONhigh and some samples of ZENhigh. Proteins enriched in DONhigh were mainly related to translation, ribosomal structure and biogenesis and cell biogenesis (Figure 4B). All differentially abundant proteins were also mapped against the KEGG database (Kyoto Encyclopedia of Genes and Genomes) to identify pathways and functional hierarchies (BRITE). Proteins could be mapped with multiple pathways or hierarchies. Broadly, the proteins were mapped to 119 KEGG orthologs (KO) and 85 pathways. Among the proteins enriched in DONhigh (cluster 2), 57.4%, 21.7%, 15.8% and 11.8% were mapped to different enzymes, ribosomes, exosomes, and transporters, while among proteins enriched in the DONlow, ZENlow and control samples, 54.2%, 11.4%, 20.0% and 25.7% were mapped to those functions, respectively.

Overall, differentially abundant proteins were mapped to the ribosome (19.3% of KO), biosynthesis of amino acids (14.2%), carbon metabolism (14.2%) glycolysis (11.7%), pyruvate metabolism (7.5%), starch and sucrose metabolism (7.5%), and pentose-phosphate (PP) pathways (5.8%). DONhigh was the treatment with the most differentially abundant proteins compared to the control group (107 proteins), followed by DONlow (8 proteins), ZENlow (3 proteins) and ZENhigh (3 proteins) (Figure S4). Only one protein, with unknown function, was differentially abundant between all the groups and the control. Proteins differentially abundant between DONhigh and control group were mostly associated with the ribosome (15 KO), carbon metabolism (13 KO), biosynthesis of amino acids (13 KO) glycolysis/gluconeogenesis (11 KO), fructose and mannose metabolism (6 KO), and pentose phosphate (PP, 6 KO) pathways. On the other hand, proteins differentially abundant among ZENhigh and the control group were associated with galactose metabolism and an unknown function.

**Figure 5:**
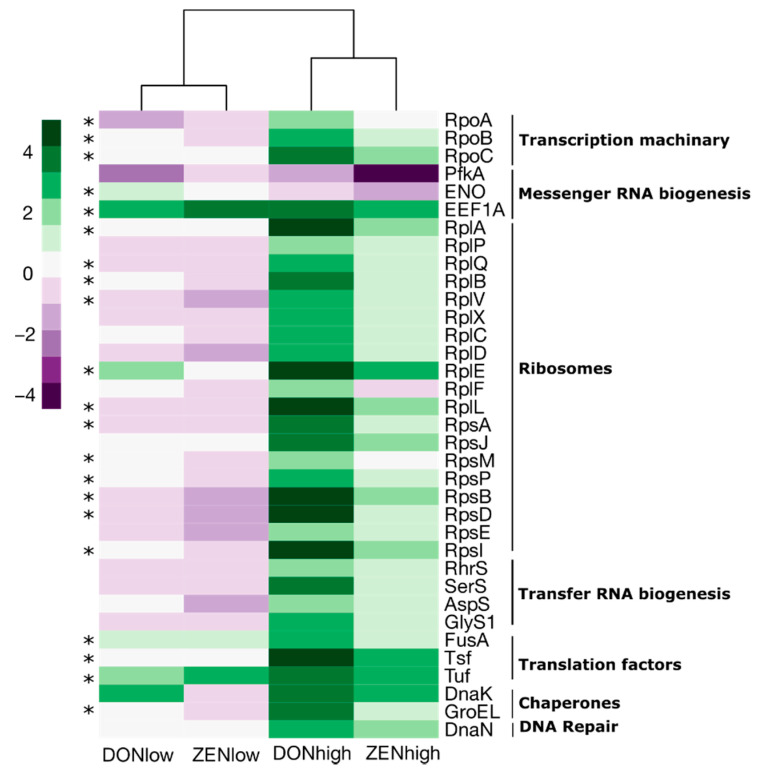
DONhigh significantly increased the abundance of bacterial proteins associated with genetic processing. Log2 fold change between each treatment and the control group. Only significantly different proteins are shown. * Differentially abundant proteins between DONhigh and control group (*p* < 0.05).

Next, differentially abundant proteins involved in genetic processing, glycolysis and PP pathways were selected and compared. Most of the proteins involved in genetic processing were ribosomal proteins (Figure 5), which were ~2–5-times more abundant in DONhigh (12 ribosomal proteins, *p* < 0.05) and ~0–2.8-times more abundant in ZENhigh (*p* > 0.05) compared to the control group. In general, the abundance of ribosomal proteins was slightly lower in DONlow and ZENlow (*p* > 0.05). Additionally, proteins of transcription, translation, chaperones, and DNA repair factors were ~2–4.8-times more abundant in DONhigh. Among all the differentially abundant proteins associated with genetic processing, only 6-phosphofructokinase 1 (Pfka) and enolase (ENO) were less abundant in DONhigh and ZENhigh compared to the control group.

**Figure 6:**
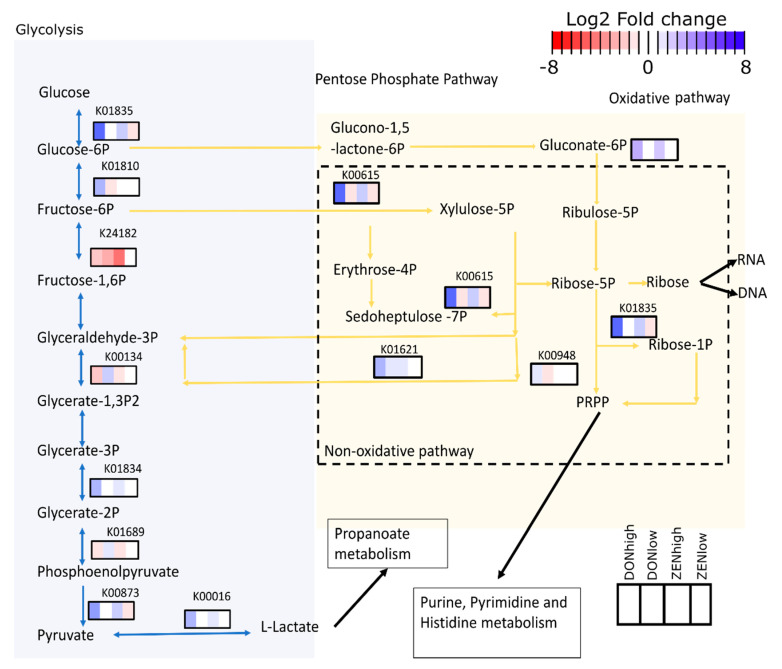
Mycotoxins reduced the abundance of key enzymes of the upper glycolysis and increased proteins of the pentose phosphate pathway. Colored rectangles represent the Log2 fold change between the treatments and the control group for each identified enzyme. KEGG orthologs (KO) identifiers are shown over each square.

The differentially abundant proteins mapped to the glycolysis and PP pathway were visualized using PathView (Figure 6). Broadly, seven enzymes associated with the glycolysis (Embden–Meyerhof pathway) were differentially abundant among the treatments. For example, the number of proteins involved in the conversion of fructose 6-phosphate to fructose 1,6-bisphosphate, glyceraldehyde 3-phosphate to glyceraldehyde 1,3-bisphosphate, and 1,3-bisphosphoglycerate to phosphoenolpyruvate was reduced both in DONhigh and ZENhigh. In contrast, proteins converting glucose to fructose were more abundant in DONhigh. Glucose 6-phosphate and fructose 6-phosphate can be redirected to the oxidative and non-oxidative PP pathway for the dissimilation of carbohydrates. Only one enzyme from the oxidative pathway was more abundant in DONhigh, while four were more abundant in the non-oxidative branch. Enzymes involved in the synthesis of ribulose 5-phosphate (oxidative) and isomerization to ribose 5-phosphate (non-oxidative) were more abundant in DONhigh. This path can lead to the biosynthesis of nucleotides and reducing agents. The oxidative decarboxylation of 6-phosphogluconate to ribulose 5-phosphate produces NADPH, which preserves the antioxidant capacities of the cell. Moreover, DONhigh and ZENhigh increased the abundance of thioredoxin-dependent peroxiredoxin 4.8- and 2.2-fold, suggesting that the mycotoxins may induce oxidative stress in gut bacteria. 

Peptides of the differentially abundant proteins between the treatments were associated mostly with Firmicutes (53.1%–79.1% across all samples), followed by Actinobacteria (0.009%–10.8%) and Proteobacteria (0.0%–0.7%). Additionally, peptides of the protein groups enriched in ZENhigh and DONhigh belonging to Actinobacteria increased from 0.3% to 4.9% and 10.8%, respectively, compared to the control, while Firmicutes decreased from 68.7% to 65.6% and 53.1%, respectively (Figure S5). This pattern is consistent with the general changes observed in the structure of the microbiome after the ingestion of the mycotoxins.

## 3. Discussion

In the present study, the effect of the mycotoxins DON and ZEN on the gut microbiome of weaned piglets was investigated using metaproteomics. Ingestion of mycotoxins can trigger negative effects on the animals as well as a response of the gut microbiome. The bi-directional interaction between the microbiome and mycotoxins can explain the microbiota modulation and removal or metabolism of the toxins [13,15]. Even though some previous studies have shown that mycotoxins alter the gut microbiome [17,18,19,20], its effect on their proteins and functions is not known. Here, changes on the weaned piglet’s gut metaproteome after 28 days of exposure at two dosages of mycotoxins were identified. Data indicated that an alteration of the gut microbiome after ingestion of DON and ZEN may be mediated by the increase in oxidative stress. Several studies have shown that mycotoxins inhibited cell viability, leading to cell arrest and apoptosis [21,22,23]. Additionally, the toxic effect has been linked to the induction of reactive oxygen species (ROS) and pro-inflammatory cytokines [24]. In the present study, a reduced abundance of proteins involved in glycolysis and an increased abundance of antioxidant agents were detected, which could indicate an adaptation of the microbiome to oxidative stress.

DONhigh and ZENhigh decreased the abundance of proteins involved in carbohydrate metabolism via glycolysis but enhanced those involved in the PP pathway. We observed that intermediate enzymes of the glycolysis, as 6-phosphofructokinase and glyceraldehyde 3-phosphate dehydrogenase, were negatively affected by the mycotoxins. These enzymes are sensitive to oxidative stress, as well as being highly regulated [25,26,27]. The conversion of fructose-6-phosphate to fructose-1,6-diphosphate is the first committed step of the glycolysis, and its inhibition allows the diversion of glycolytic intermediates onto the PP pathway [28,29]. DONhigh and ZENhigh increased the enzyme abundance from both the oxidative and non-oxidative branch of the PP pathway. The PP pathway is considered as the first line of defence when undergoing oxidative stress [25,28,30]. The conversion of glucose 6-phosphate to ribulose 5-phosphate reduces NADP+ into the reducing agent NADPH. NADPH is key in maintaining the antioxidant capacities of the cell. The enhanced abundance of proteins involved in the interconversion of gluconate 6-phosphate to ribulose 5-phosphate due to high dosages of DON and ZEN could be caused by the ongoing oxidative stress. Moreover, the enhanced abundance of the non-oxidative-PP pathway proteins may offer the possibility for the formation of essential precursors for the synthesis of peptidoglycan, aromatic amino acids, RNA and DNA. Those are especially important in response to deep damage of the cellular structure [31].

Even though the abundance of proteins involved in the conversion of glycolytic intermediates was reduced, several enzymes of the lower glycolysis were significantly more abundant in DONhigh and ZENhigh. The PP pathway can function as a bypass to produce pyruvate instead of the glycolysis [25,32]. Moreover, the observed shifting between the glycolysis and the PP pathway could also be the consequence of the altered microbial diversity. It has been shown that bacteria lacking key enzymes for glycolysis and those who favour the oxidative-PP can cope with oxidative stress, while those that favour glycolysis cannot [28]. 

The gut microbiome produces antioxidants to deal with the oxidative stress after the ingestion of mycotoxins. DONhigh and ZENhigh increased the abundance of thioredoxin-dependent peroxiredoxin between 1.5- and 4.8-fold. The antioxidant enzyme peroxiredoxin plays an important role in the defence against oxidative stress of bacteria like *Corynebacterium*, *Cyanobacterium* and *Neisseria* [33,34,35]. This enzyme is a thiol-specific antioxidant that can detoxify hydrogen peroxide, alkyl peroxides, and peroxynitrite. Moreover, peroxiredoxin can transform ZEN into small estrogenic metabolites with an unknown structure [36,37]. This could indicate that peroxiredoxin acts in both ways; firstly it copes with the indirect oxidative stress produced by the toxin, and secondly, it acts as a detoxifier of the mycotoxin. Additionally, the potential of the pig gut microbiome to transform ZEN was previously shown in in vitro experiments [38].

Ribosomal proteins and other translation and transcription elements were more abundant in the mycotoxin groups, such as 19 ribosomal proteins which increased in abundance in DONhigh and ZENhigh. Ribosomal proteins such as Rpsl, RpsR and RpsT were shown to be highly regulated by the extremophile *Deinococcus radiodurans* during oxidative stress [39]. It was hypothesized that damaged proteins need to be rapidly replaced when *D. radiodurans* is under oxidative stress. Interestingly, a concomitant increased abundance of the chaperones DnaK and GroEL was identified in the present study. In stressful conditions, DnaK interacts with ribosomal proteins and functions as a stabilizer for the subsequent folding by GroEL [40,41]. This may indicate that during oxidative stress triggered by mycotoxins, the gut microbiome requires fast regulation of translation and protein repair.

The gut microbiome is altered after the ingestion of mycotoxins. Previous studies have found that ZEN and DON have a general negative effect on the gut microbial diversity of pigs, mice, rabbits, and broilers [15]. Our data showed that proteins of the bacterial phylum Firmicutes decreased, while proteins of Actinobacteria increased due to DONhigh and ZENhigh, which is consistent with previous reports on pigs [17]. Furthermore, peptides of proteins derived from Actinobacteria and associated with microbial metabolism, genetic processing and oxidative stress increased in ZEN and DON, indicating a possible adaptation of this group to the stress caused by the mycotoxins. The dysbiosis of the gut microbiome is probably associated with the gut barrier disruption and not a direct antimicrobial effect [15,42]. For example, increased redox conditions in the gut can deplete strict anaerobes such as some Firmicutes (*Eubacteriaceae*, *Lachnospiraceae*, *Ruminococcaceae* and *Erysipelotrichaceae*), which was observed in this study [43]. On the other hand, some human gut bacteria such as *Bifidobacterium* can metabolize and immobilize (modified) mycotoxins [15,42]. DON and ZEN increased the abundance of proteins assigned to the family *Bifidobacteriaceae* in our samples. Additionally, it has been reported that the response of the gut microbiome varies based on the gut section [17,44], but this effect was not observed in our study as the metaproteome profile was similar between the ileum and jejunum.

## 4. Conclusions

In summary, the present study reinforces the idea that the alteration of the gut microbiome due to the ingestion of mycotoxins can be associated with an increase in oxidative stress. The reduced abundance of enzymes sensitive to oxidative stress and the increase in proteins able to cope with it are evidence of this. Finally, different strategies should be designed to tackle the damage caused to the gut and microbial cells during the increase in ROS and inflammatory compounds and proteins.

## 5. Materials and Methods

### 5.1. Animal Experiment and Sampling

In total, 50 female weaned piglets (*Sus scrofa domestica,* Large White × Piétrain) were included in the trial and kept in the stables of the University Clinic for Swine, Vetmeduni Vienna. The animals derived from the university’s own piglet production at the VetFarm Medau in Lower Austria. Moving of the animals to the campus of the University of Veterinary Medicine and regrouping took place on the day of weaning at the age of four weeks (day 7). After delivery, the animals were assigned into five groups (10 animals per group). Each group was housed in a separate pen with straw bedding. Straw was previously tested to be free from mycotoxins. Food and water were fed ad libitum during the entire testing period. Prior to feeding with mycotoxin containing diets, weaned piglets underwent an acclimatization phase (day 7 to 0) and were adapted to weaned piglets feed 1 (Figure 1). Before changing to weaned piglets feed 2 on day 14, weaned piglets feed 1 was blended with weaned piglets feed 2 for one week (day 7 to day 14) (Figure 1). Both diets (weaned piglets feed 1 and weaned piglets feed 2) are standard weaned piglet diets (BIOMIN Holding GmbH, Austria). In the mycotoxin exposition phase (day 0 to day 28), the control group was fed the mycotoxin-free base diet. For artificial contamination of diets, culture material of different *Fusarium graminearum* strains, containing either 1.36 mg/kg ZEN or 0.87 g/kg DON, were used (BiMM—Bioactive Microbial Metabolites Group, Universitäts und Forschungszentrum, Tulln, Austria). Used strains specifically produce either DON or ZEN, which excluded co-contamination of the diets. To ensure the homogeneous distribution of mycotoxins, premixes with inulin were prepared, which were mixed into the basal feed at an inclusion rate of 1.4%. Final mycotoxin concentrations were assessed via high performance liquid chromatography-mass spectrometry analysis (Romer Labs GmbH, Austria). The results show average ZEN contamination levels of 0.01 µg/kg, 679 µg/kg and 1623 µg/kg in the feed for the control, ZENlow and ZENhigh groups, respectively, while the DON levels in the feed of the control, DONlow and DONhigh groups were 61 µg/kg, 870 µg/kg and 2493 µg/kg. Natural contamination of diets with other major mycotoxins was marginal (fumonisin B1 90 µg/kg) or absent (aflatoxin B1, ochratoxin, ergot alkaloids).

Weaned piglets were checked daily for overall health. Body weight was measured on days −7, 0, 7, 14, 21 and 28 before necropsy (Figure 1). Weaned piglets were anesthetized (Stresnil^®^, Elanco Animal Health, Bad Homburg, Germany and Narketan^®^, Vétoquinol Österreich, Vienna, Austria) and euthanized (T61^®^, Intervet, Vienna, Austria) on day 28 of mycotoxin exposure. For this metaproteomics study, out of the 50 initial animals, only three weaned piglets per group (15 in total) were randomly selected and sampling was conducted as follows: after opening the visceral cavity, the duodenum and rectum were clamped and the intestine was removed from the visceral cavity. The jejunum and ileum were separated by clamping to avoid mixing of digesta. Digestal and mucosal content was collected from both sites. The mucosa was rinsed with sterile ice-cold phosphate buffered saline if digesta content was left. Samples were immediately placed on ice and stored at −80 °C for further analysis. In total, 60 samples were collected (15 weaned piglets, four matrices: jejunum and ileum, digesta and mucosa samples).

### 5.2. Sample Preparation

A homogenous sample mixture was prepared by mixing 500 mg of sample with 15 mL of washing buffer (50 mM sodium phosphate, 0.1% Tween 80, pH 8). Cell solubilization was performed in a sonication bath for 10 minutes and subsequent agitation (20 min, 100 oscillations/min) at room temperature and centrifugation as described in Tilocca et al. (2016) [45]. The resulting pellet was resuspended in 200 µL of extraction buffer (20 mM Tris-HCl, 2% SDS, pH 7.5) and heated to 60 °C for 10 min and centrifuged at 1400 rpm. Further, one milliliter of Tris-HCl buffer was added (20 mM Tris-HCl, 0.1 mg/mL MgCl_2_, 1 µL/mL Benzonase, 1 mM PMSF) and the proteins were extracted using a sonication probe (5 cycles for 1 min, 60% amplitude for 0.5 cycle). The samples were reheated in Thermomixer at 37 °C for 10 min and then centrifuged (10,000 *g*, 10 min, 4 °C). The supernatant was stored at −20 °C until further use [46]. The Bradford assay was used for protein quantification measured at Lambda 595 nm. Briefly, 595 µL of Bradford was mixed with a 5 µL sample and incubated for 5 minutes. Protein extracts with at least 100 µg/mL proteins were precipitated using acetone (5 volumes acetone and 1 volume sample) overnight at 4 °C, centrifuged (14,000 *g*, 30 min, 4 °C) and dried using speed vac [47]. Samples were pre-fractioned and separated from remaining contaminants using SDS-Page [48,49,50]. In-gel digestion was performed overnight at 37 °C using Trypsin (Promega, Madison, WI, USA) [51]. The peptide pellet was mixed with 110 µL of 0.1% formic acid and bound using five C18 layers in a Stage Tip and washed using acetic acid and acetonitrile. 

### 5.3. LC–MS/MS Analysis

Peptide mixtures were measured using a Q-Exactive HF-X mass spectrometer (Thermo Fisher Scientific, Darmstadt, Germany) faced with an EasyLC 1000 nano-UHPLC (Thermo Fisher Scientific, Darmstadt, Germany). Separation of peptides was performed on a 20 cm fused silica column of 75 μm inner diameter (Proxeon Biosystems, Odense, Denmark). The column was in-house packed with reversed-phase ReproSil-Pur 120 C18-AQ 1.9 μm resin (Dr. Maisch GmbH, Ammerbuch, Germany). Peptides were loaded onto the column in solvent A (0.1% formic acid) at a flow rate of 500 nL/min and subsequently eluted with an 87 min segmented gradient of 10%–50% HPLC solvent B (80% ACN in 0.1% formic acid). The MS/MS instrument was set to positive ion mode. Full scans were acquired in the mass range from m/z 300 to 1650 in the Orbitrap mass analyser at a resolution of 120,000 followed by HCD fragmentation of the 12 most intense precursor ions. High-resolution MS/MS spectra were acquired with a resolution of 30,000. The target values were 3 * 106 charges for the MS scans and 1 × 105 charges for the MS/MS scans with a maximum fill time of 25 and 45 ms, respectively. Fragmented masses were excluded for 30 s after MS/MS. Spectra de-noising was performed prior to peptides identification by considering only the top 12 peaks in a window of 100 Da width.

### 5.4. Data Analysis

Identification and quantification of peptides/protein groups and their taxonomic assignment and functional annotation was done using MetaLab v.2.1.0 [52]. Host protein identification was based on a reference proteome of *Sus scrofa* (Uniprot: UP000008227), which is composed of 22,168 genes. Similarly, the gut microbiome protein composition was identified using the “catalogue of the pig gut microbiome” composed of 7.7 million genes representing 719 metagenomic species [53]. The sample-specific database construction by spectral clustering strategy implemented in MetaLab was used. After, identified peptides and proteins were filtered based on FDR 0.01 and protein quantification was obtained with MaxqQuant maxLFQ algorithm implemented in MetaLab. Carbamidomethyl (C) was used as mixed modification, and Oxidation (M) and N-terminal acetylation as variable modifications. Additionally, the option “matching between runs” was used and the instrument resolution was set as “High-High.” 

LFQ-intensities of the identified protein groups were Log_2_(x+1) transformed and normalized by a quotation transformation (x/mean) using the R packages ‘clusterSim’. Transformed and normalized data were visualized using an unsupervised non-linear dimensionality reduction algorithm, t-distributed stochastic neighbor embedding (tSNE), perplexity = 10, maximum iteration = 1200. Dissimilarity between the samples was calculated using the Bray–Curtis dissimilarities. 

Peptide sequences were taxonomically assigned to the lineage of the lowest ancestor (LCA) and mapped to the pep2tax database as previously reported [52,54]. Eukaryota, bacteria, archaea and viruses were included in the calculations. Relative abundance was calculated by summing the LFQ intensities of all found taxa. Additionally, the relative abundance of the phyla Actinobacteria and Firmicutes was used to calculate the ratio between those groups across all samples.

### 5.5. Statistical Analysis

Statistical tests were performed in R base v.4.0.2 (Libraries: ‘car’ and ‘vegan’) [55,56] and LFQ-Analyst [57]. Normal distributed data was compared using ANOVA and Tukey’s test, while Kruskal–Wallis and Wilcoxon rank-sum tests were used for non-normal distributed data. Differences between protein group composition were evaluated using LFQ-Analyst. Data were imputed following the Perseus approach. Changes between the proteins were considered significant if *p* < 0.05, Log_2_ fold change >2, and >1 peptides were identified. *p* values were corrected by Benjamini–Hochberg. Variance associated with the treatments or gut-section were evaluated by the PERMANOVA test (vegan package) based on Bray–Curtis dissimilarity.

### 5.6. Data Visualization

Box Plots, heatmaps and t-SNE plots were visualized using R base v.4.0.2 (Libraries: ‘ggplot’, ‘pheatmap’ ‘viridis’ and ‘RColorBrewer’). Additionally, heatmaps of the significantly different protein groups among the treatments clustered by groups were visualized using LFQ-Analyst [57]. Metabolic pathways were reconstructed with KEGG Mapper [58] and Pathview [59].

## Data Availability

All raw data, mass spectrometry proteomics, have been deposited to the ProteomeXchange Consortium via the PRIDE partner repository (http://www.proteomexchange.org/) with the dataset identifier PXD027185.

## Supplementary Materials

The following are available online at https://www.mdpi.com/article/10.3390/toxins13080583/s1. Table S1. Summary of the identified peptides and protein groups. % MS/MS spectra identified as peptide, at a 1% FDR; Figure S1. Relative abundance at phylum level of the gut microbiome after the ingestion DON and ZEN; Table S2. Relative abundance (%) at phylum level of the gut microbiome after the ingestion of DON and ZEN; Table S3. Significant differences of the relative abundance at phylum level of the gut microbiome after the ingestion of DON and ZEN; Figure S2. The number of proteins identified by COG categories. Only differentially abundant proteins between the treatments are shown; Figure S3. Pearson correlation of the samples based on the differentially abundant protein groups; Figure S4. Number of differentially abundant proteins compared to the control and their respective overlap between the treatments; Figure S5. Taxonomic distribution of the peptides enriched in DONhigh and ZENhigh across all samples.

## Abbreviations

DON	deoxynivalenol
ZEN	zearalenone
BW	Body weight
PP	Pentose phosphate
KO	KEGG orthology
