# Supplementary material for: Metaproteomics Reveals Alteration of the Gut Microbiome in Weaned Piglets Due to the Ingestion of the Mycotoxins Deoxynivalenol and Zearalenone
Source: Toxins (Basel). 2021 Aug 21;13(8):583. doi: 10.3390/toxins13080583 (PMC8402495; doi:10.3390/toxins13080583)
Supplement: Supplementary file 1 [file toxins-13-00583-s001.zip › toxins-1321411-supplementary.pdf]

Supplementary Materials: Metaproteomics Reveals Alteration of the Gut Microbiome in Weaned Piglets Due to the Ingestion of the Mycotoxins Deoxynivalenol and Zearalenone

Johan S. Sáenz, Alina Kurz, Ursula Ruczizka, Moritz Bünger, Maximiliane Dippel, Veronika Nagl, Bertrand Grenier, Andrea Ladinig, Jana Seifert and Evelyne Selberherr

Table S1. Summary of the identified peptides and protein groups. % MS/MS spectra identified as peptide, at a 1% FDR.

Sample	Microbiome			*Sus scrofa*		
	Peptide sequences	MS/MS%	Protein groups	Peptide sequences	MS/MS%	Proteins groups
**Digesta**	9496	3.2	1427	2082	2.1	193
**Mucosa**	876	1.5	165	4748	6.2	309

Table S2. Relative abundance (%) at phylum level of the gut microbiome after the ingestion of DON and ZEN.

**Treatment**	**Phylum**									
	**Actinobacteria**	**Archaea**	**Bacteria**	**Bacteroidetes**	**Candidatus Woesebacteria**	**Cyanobacteria**	**Euryarchaeota**	**Firmicutes**	**Planctomycetes**	**Proteobacteria**
**ZENlow**	0.3903	0.0005	24.8684	0.9168	0.0146	0.0000	0.0032	73.5198	0.0000	0.2864
**Control**	1.1933	0.0006	22.8566	0.0240	0.0003	0.0027	0.0038	75.1500	0.4289	0.3397
**DONlow**	0.1430	0.0012	24.7747	0.0069	0.0000	0.0000	0.0073	74.8987	0.0000	0.1682
**DONhigh**	14.9615	0.0004	24.9569	0.2057	0.0050	0.0000	0.0024	59.8144	0.0000	0.0537
**ZENhigh**	8.2868	0.0000	23.4383	0.0184	0.0067	0.0000	0.0000	67.8083	0.0000	0.4415

Table S3. Significantly differences of the relative abundance at phylum level of the gut microbiome after the ingestion of high and low dosage of mycotoxins. Kruskall-Wallis and Wilcoxon rank sum test, p values were corrected by Benjamini-Hochberg in multiple comparisons. +Comparison against all and *comparison against control group.

Phylum	Treatment				
	All+	DonHigh*	DonLow*	ZenHigh*	ZenLow*
Actinobacteria	0.0004	0.0137	> 0.05	> 0.05	> 0.05
Unassigned Archaea	> 0.05	> 0.05	> 0.05	> 0.05	> 0.05
Unassigned Bacteria	> 0.05	> 0.05	> 0.05	> 0.05	> 0.05
Bacteroidetes	0.0221	> 0.05	> 0.05	> 0.05	> 0.05
Candidatus Woesebacteria	> 0.05	> 0.05	> 0.05	> 0.05	> 0.05
Cyanobacteria	> 0.05	> 0.05	> 0.05	> 0.05	> 0.05
Euryarchaeota	> 0.05	> 0.05	> 0.05	> 0.05	> 0.05
Firmicutes	0.0037	0.0081	> 0.05	> 0.05	> 0.05
Planctomycetes	<.0001	0.0150	0.0150	0.0150	0.0150
Proteobacteria	> 0.05	> 0.05	> 0.05	> 0.05	> 0.05

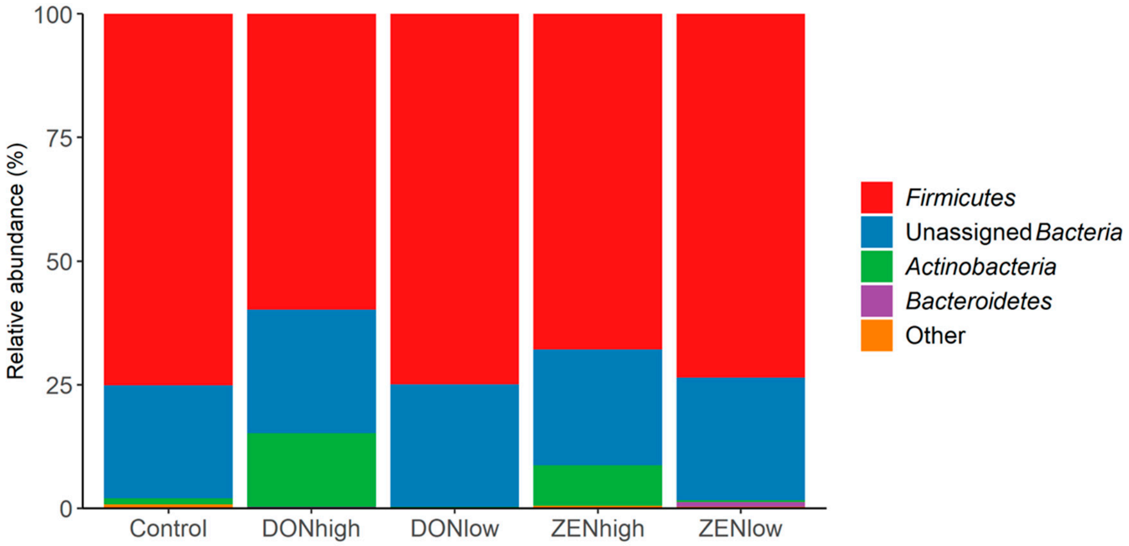

Figure S1. Relative abundance at Phylum level of the gut microbiome after the ingestion DON and ZEN.

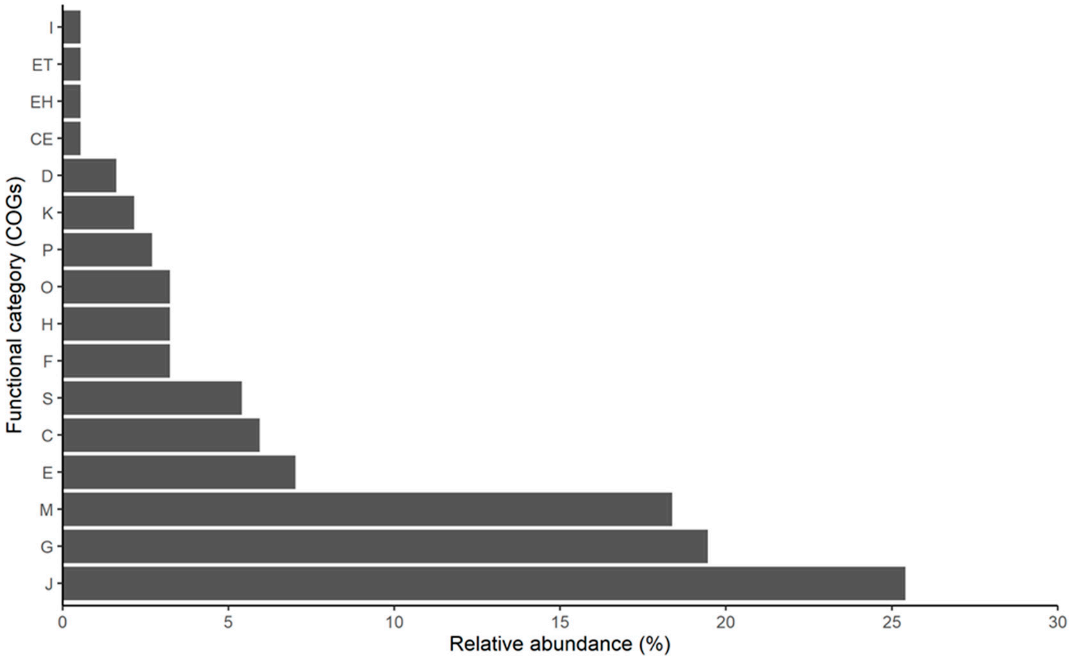

Figure S2. The number of proteins identified by COG categories. Only differentially abundant proteins between the treatments are shown. [J] Translation, ribosomal structure and biogenesis, [G] Carbohydrate transport and metabolism, [M] Cell wall/membrane/envelope biogenesis, [S] Function unknown, [E] Amino acid transport and metabolism, [C] Energy production and conversion, [F] Nucleotide transport and metabolism, [H] Coenzyme transport and metabolism, [O] Post-translational modification, protein turnover, and chaperones, [K] Transcription, [D] Cell cycle control, cell division, chromosome partitioning, [P] Inorganic ion transport and metabolism, [L] Replication, recombination and repair, [I] Lipid transport and metabolism

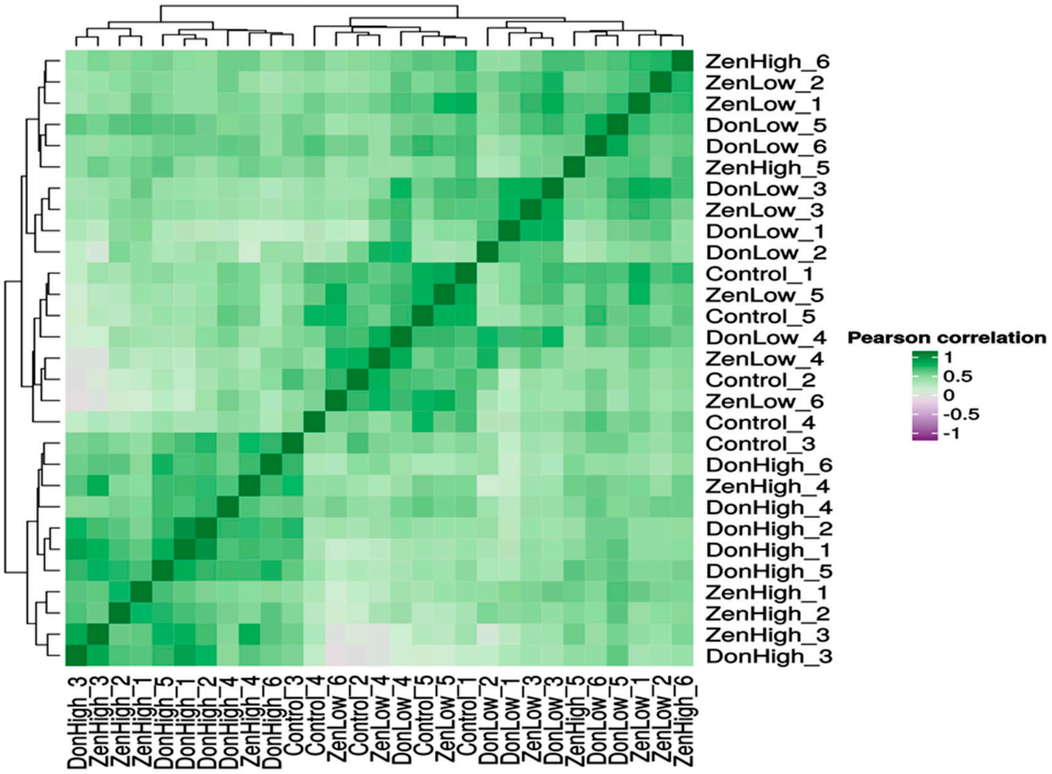

Figure S3. Pearson correlation of the samples based on the differentially abundant protein groups.

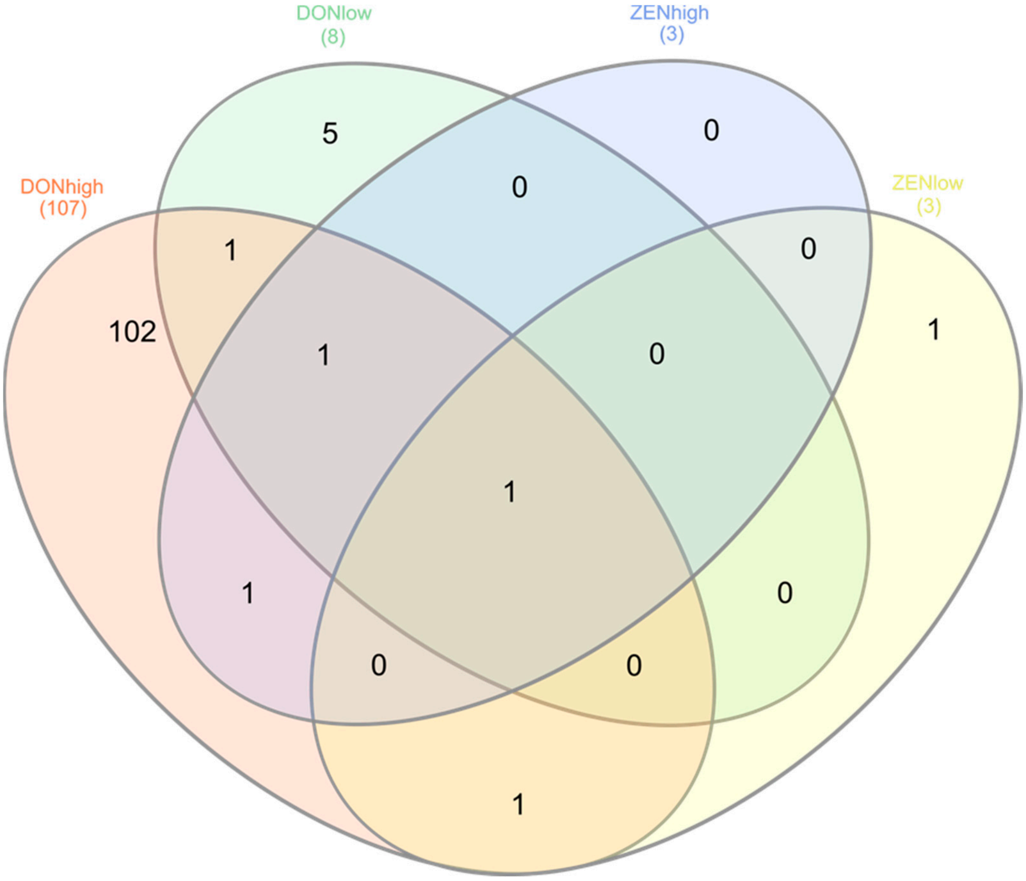

Figure S4. Number of differentially abundant proteins compared to the control and their respective overlap between the treatments.

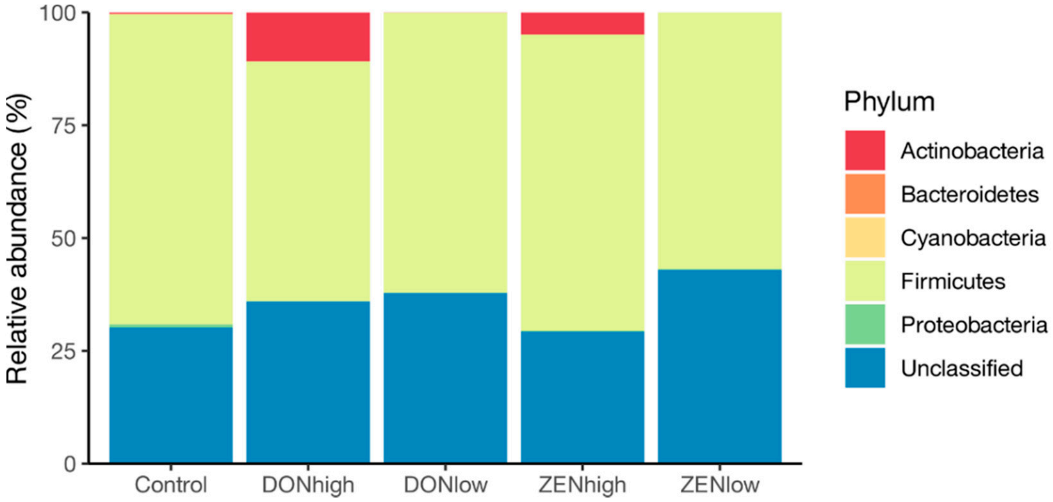

Figure S5. Taxonomic distribution of the peptides enriched in DONhihg and ZENhigh across all samples.
